# Nanomedicine of tyrosine kinase inhibitors

**DOI:** 10.7150/thno.48662

**Published:** 2021-01-01

**Authors:** Veronika Smidova, Petr Michalek, Zita Goliasova, Tomas Eckschlager, Petr Hodek, Vojtech Adam, Zbynek Heger

**Affiliations:** 1 Department of Chemistry and Biochemistry Mendel University in Brno, Zemedelska 1, 613 00 Brno, Czech Republic.; 2 Central European Institute of Technology, Brno University of Technology, Purkynova 656/123, 612 00 Brno, Czech Republic.; 3 Department of Paediatric Haematology and Oncology, 2nd Faculty of Medicine, Charles University, and University Hospital Motol, V Uvalu 84, Prague 5 CZ-15006, Czech Republic.; 4 Department of Biochemistry, Faculty of Science, Charles University, Albertov 2030, 128 40 Prague 2, Czech Republic.

**Keywords:** Bioavailability, Drug delivery, Nanotechnology, Targeted therapy

## Abstract

Recent progress in nanomedicine and targeted therapy brings new breeze into the field of therapeutic applications of tyrosine kinase inhibitors (TKIs). These drugs are known for many side effects due to non-targeted mechanism of action that negatively impact quality of patients' lives or that are responsible for failure of the drugs in clinical trials. Some nanocarrier properties provide improvement of drug efficacy, reduce the incidence of adverse events, enhance drug bioavailability, helps to overcome the blood-brain barrier, increase drug stability or allow for specific delivery of TKIs to the diseased cells. Moreover, nanotechnology can bring new perspectives into combination therapy, which can be highly efficient in connection with TKIs. Lastly, nanotechnology in combination with TKIs can be utilized in the field of theranostics, i.e*.* for simultaneous therapeutic and diagnostic purposes. The review provides a comprehensive overview of advantages and future prospects of conjunction of nanotransporters with TKIs as a highly promising approach to anticancer therapy.

## Introduction

With the incidence of 18.1 million new cases worldwide and death count reaching 9.6 million people in the year of 2018, cancer is a serious player on the field of human health 1. Tyrosine kinases (TKs) are important targets for cancer treatment due to their role in signal transduction and subsequent cell proliferation that can turn into neoplastic cell transformation and tumor growth 2. Inhibitors of these kinases are commonly used in the treatment of non-small cell lung cancer (NSCLC), chronic myeloid leukemia (CML) and some cases of breast and colorectal cancer, advanced gastrointestinal stromal tumor, renal cell, hepatocellular or thyroid carcinoma and soft tissue sarcoma 3.

Tyrosine kinase inhibitor (TKI) therapy, however beneficial, has its limitations and disadvantages. Off-target activity causes multitude of adverse events, negatively affecting the quality of patient's life and continuation of therapy 2. Moreover, cancer tends to develop variety of mechanisms of acquired drug resistance, resulting in inefficacy of TKI medications. However, in recent years, more and more publications focus on the incorporation of these small molecule drugs into nanomedicinal applications in order to facilitate progress in cancer therapy 4.

This review is focused on two particular themes, concerning TKIs and nanomedicine. In its first part, we indulge in the matters of TKs and their small molecule inhibitors. Second part is dedicated to nanoformulations of TKIs and to the potential nanomedicine has to help overcome the limitations of TKI therapy and to improve it.

## Tyrosine kinases

TKs are transmembrane (receptor) or unattached (non-receptor) enzymes that transfer γ‑phosphate from adenosine triphosphate (ATP) to tyrosine residue of a substrate molecule. This transfer causes allosteric or functional change of the protein and represents an essential mechanism for activation and direction of cell signaling 5. There are ~90 members of protein TK family, 58 of which are receptor TKs (RTKs), and 32 are non-receptor TKs. Moreover, these two groups can be divided - according to Robinson *et al.* (2000) - into 20 and 10 subfamilies, respectively, based on kinase domain sequence 6, although designation and number of subfamilies can be variable. The 20 subfamilies of RTKs can be grouped according to the typology of the receptor. There are two important oncotargeted groups based on receptors of type I (EGFR/HER, alternate Cys and Leu rich domains) and type IV (VEGFR, Ig-like domains). In addition, there are two groups with influence on neoplastic growth, although not yet oncotargeted - type II Insulin receptor family (Leu-Cys-Leu domains attached with fibronectin) and type IX Ephrins (Ig-like + Cys-rich + two fibronectin domains). Lastly, there is a group of miscellaneous kinases with different (e.g. ALK, DDR, MET, RON, RYK) or truncated extracellular domains (LMR, STYK1, etc.) 5.

### RTKs structure

RTKs are membrane glycoproteins that transfer signals from extracellular into intracellular environment. Therefore, their structure consists of an extracellular hydrophilic domain, a hydrophobic transmembrane segment and an intracellular domain. Extracellular part of RTKs can be very variable according to their corresponding ligand. Binding of the ligand needs to be specific and reversible. A number of low-energy bonds like hydrogen, ionic, hydrophobic or Van der Waals interactions are involved 7. Intracellular part, however, is quite the opposite, consisting of a juxtamembrane and a TK domain with a flexible *C*-terminal tail. In some cases, TK domain can be divided in two parts by a kinase insertion 5. ATP binding site, situated on the *N*-terminal lobe, has a conserved sequence, in which a loop with Gly residue serves to localize phosphate on the ATP molecule 8.

### RTKs activation and activity

RTKs are mostly monomeric, undergoing homo- or heteromerization even before or upon ligand binding 9. In the absence of ligand, RTKs show a basal kinase activity, possibly due to self- or exophosphorylation 5. Mayer proposes a dynamic phospho-turnover model where constant autophosphorylation and phosphatase activity maintains a stable background “noise” signal 10. Binding of the ligand causes the intracellular part of juxtaposed kinase domains to transphosphorylate juxtamembrane and kinase domains with *C*-terminal tail. This fully activates the RTK 5. Another Mayer's suggestion envisions a dynamic ligand binding in a form of “hopping” from domain to domain, consequently activating up to 20 RTKs in the process 10. Inhibition of kinase activity is executed by various mechanisms, such as dephosphorylation by protein phosphatases, endocytosis or ubiquitination 5. Gelens *et al*. further extend the dynamic theory, showing that even when the signal seems to be stably switched on, there are rapid phosphorylation-dephosphorylation cycles happening 11.

After the RTK is activated, Src homology-2 (SH2) and phosphotyrosine-binding (PTB) domains of cytoplasmic proteins bind to the phosphorylated tyrosine to initiate the signaling cascade. These adaptive (SH2 containing) and anchoring (PTB containing) proteins have either intrinsic enzymatic activity themselves (Src, PLCγ, GAP, PTPN11, *etc*.) or recruit other enzymes, which are involved in various signaling pathways (Nck, Crk, Shc, Grb2, *etc*.) that lead to growth stimulation, survival, migration, and actin reorganization 12.

### Roles of TKs in cancer

TKs and their phosphorylation mechanism are in control of four essential signaling networks mediating cancerogenesis, cancer progression and maintenance. These networks affect proliferation, viability, motility and cytostasis or differentiation. In detail, this entails upregulation or constitutive activation of proliferative signals, downregulation of cell death signals and maintenance of microenvironment conditions to favor tumor progression and diffusion 13.

Fleuren *et al.* created a consensus of 91 PKs out of 1,100 cancer driver genes, compiled from multiple pan-cancer studies. TKs stand for 40 % of these PKs; 36 of all 58 known RTKs being represented among them 14. Both, the kinase catalytic domain and the “gatekeeper” residue in *C*‑terminal part, which controls access to ATP-binding pocket, are frequently involved in mutations. Genome-wide amino acid substitutions including Arg, Cys, Trp, Tyr and substitutions in the catalytic domain involving Gly and Asp make PKs more prone to developing a disease. In addition, virtually all organs are affected by mutations in PKs 13.

**Figure 1:**
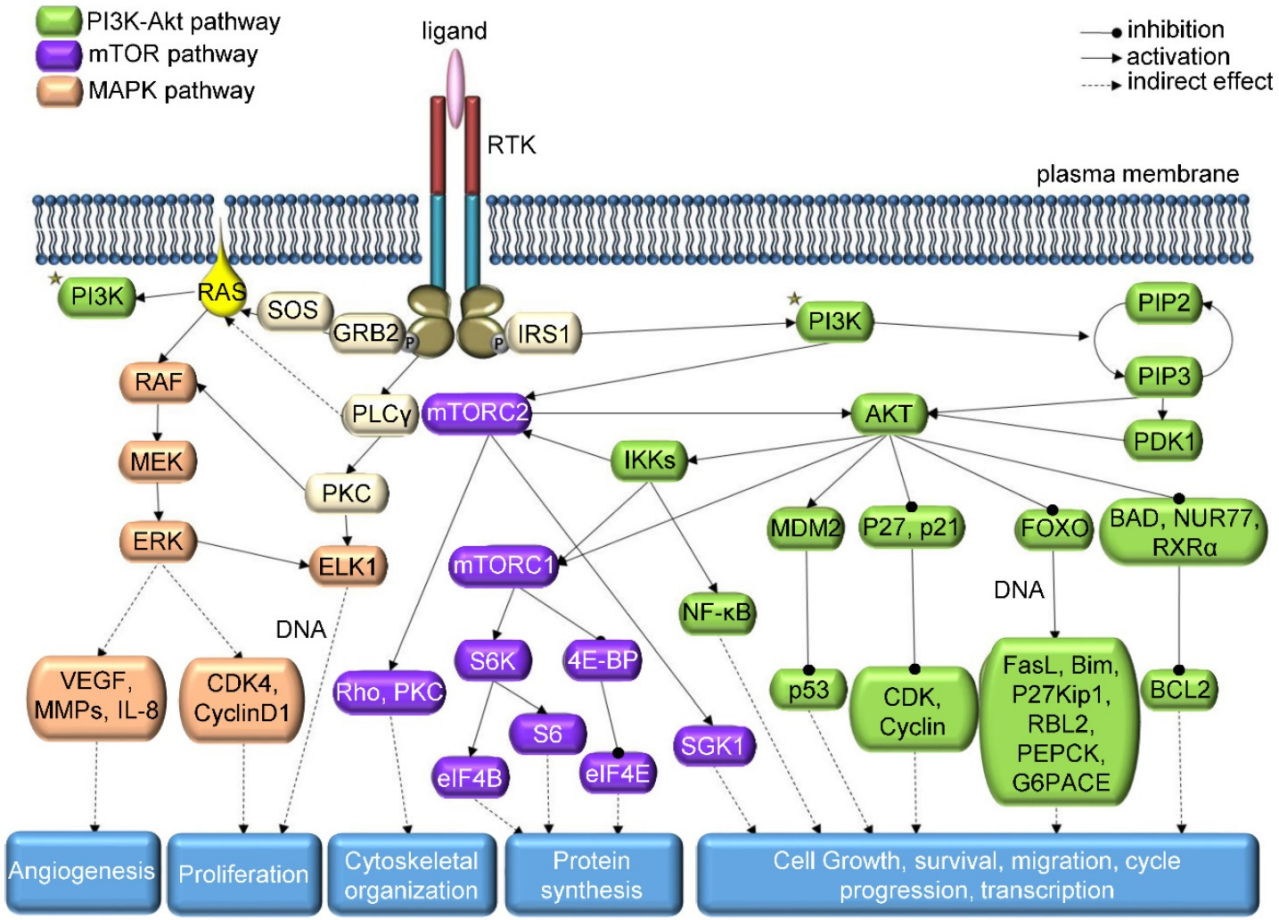
Schematic representation of RTK downstream signaling - modified from KEGG: Kyoto Encyclopedia of Genes and Genomes Database.

In the elaborate network of TK signaling (**Figure 1**), consisting mainly of MAPK, PI3K/AKT, Src, PLCγ and JAK/STAT pathways 7, not only genetic mutations are responsible for abnormal level of kinase activity; chromosomal translocations, overexpression or autocrine activation may also play this role 15. On the other hand, for TKs to be considered an oncogenic agent, it is not necessary for their function to be altered. Cancer tissue can benefit even from a perfectly normal kinase activity; therefore, oncogenic kinase is a term encompassing more than just kinases derived from oncogenic genes 13. However, the streamlining function of other kinases, phosphorylation-dephosphorylation cycle, internal molecular control by regulatory domains or subunits and local subcellular segregation of single kinases may be disrupted during oncogenesis and may cause overactivity 5. Lahiry *et al*. scanned 67 germline disorders related to kinase function, identifying 50 causative kinases, approximately half of them being TKs 16. More than 80% of the 915 detected mutations were located in the catalytic kinase domain gene or in its close proximity. In addition, a study of 3,000 cancer tissue samples from 12 tumor types revealed that most mutations were present in common oncogenic pathways RTK/RAS/RAF, PI3K/AKT/mTOR, cell cycle and p53-DNA repair, with the first route being affected in about half of the tumor samples 17.

Each TK has its own specific function that can become a factor in the emergence of neoplastic growth and progression. For instance, EGFR is especially important in NSCLC. Many EGFR mutations co-occur with other genetic alterations (e.g. mutations in TP53, PIK3CA, BRAF, MET, etc.), causing tumor progression and therapy resistance 18. EGFR overexpression is also common in colorectal cancer 19, 20 and phosphorylation of EGFR is considered a good prognostic biomarker in squamous cell carcinoma 21. In genomic characterization of 91 aberrant out of 206 glioblastoma samples, EGFR alterations were found in 45% of cases, with 24.0% amplifications, 17.5% amplifications with point mutations and 3.3% point mutations 22.

Next generation sequencing of 4,853 tumors revealed involvement of FGFR aberrations in 7.1% of samples, mostly in urothelial, breast, endometrial, squamous lung and ovarian cancers - gene amplifications being the most frequent aberration involved 23. In urothelial bladder cancer RNA sequencing study, RTKs were amplified, mutated or fused in 45% of samples, 15% of which had altered FGFR3 24. In brain metastatic breast cancer, FGFR1 was more frequently altered than other FGFR family members, and patients with this cancer type had more FGFR aberrations than non-brain metastatic breast cancer patients 25.

VEGFR has complex signaling affairs with both immune and cancer cells 26. VEGFR-1 supports tumor angiogenesis, progression and invasiveness 27. In obese mouse model, abrogation of VEGFR-1 expression normalized tumor immune environment phenotype and inhibited obesity-induced tumor progression 28. Recently, it was found to be highly expressed in glioblastoma tissue, showing ~30% higher expression than in surrounding parenchyma tissue 29. VEGFR-2 is expressed in breast cancer 30, NSCLC and other cancer types 31; it promotes tumor angiogenesis and cancer cell survival through autocrine/paracrine VEGF/VEGFR-2 loop.

PDGFR impacts tumor development by affecting fibroblasts, vascular and cancer cells 32. Alterations in PDGFR were found in approx. 13% of glioblastoma patients 22. In gastrointestinal stromal tumor sequencing, 12% of samples harbored PDGFR mutations, and 78% of samples contained KIT gene mutations 33. In prostate cancer, PDGFR-β was discovered to have higher expression in tumor stroma rather than in normal stroma and this expression was associated with disease recurrence 34.

In CML, fusion of chromosome 22 and 9 gives rise to chimeric Bcr-Abl protein, where Abl kinase domain is constitutively activated and drives the disease progression 35. Src kinase was found to be overexpressed in liver 36 and breast cancer 37, which had mitigating effect on mitochondrial activity, and consequently supported tumor progression and metastasis. *MET* and *HER2* gene amplifications are considered important driver oncogene alterations in lung adenocarcinomas 38. HER2 amplification is also significantly present in breast cancer 39, with the rate of about 15-30%, and is also found in 10-30% of gastric or gastroesophageal cancers 40. These were but a few examples of TKs involved in various types of cancers. Researchers have discovered many more over the decades of vigorous investigation, and thus are still markedly extending the number of druggable targets suitable for development of novel therapeutic modalities.

## Small molecule inhibitors of TKs (TKIs)

From a drug discovery point of view, inhibition of PK activity can be achieved by a molecule that binds either the ligand or the PK itself to prevent ligand binding, dimerization or catalytic activity 41. Alternatively, some drugs have been developed to cause kinase degradation 42. Antibodies can bind either PK's ligand or extracellular domain 41. However, they are only weak antagonists, as activating mutations in kinase domains are not inhibited by them 15. Small-molecule inhibitors target ATP‑binding site, depriving kinases of phosphate, or induce allosteric changes in low sequence homology portions of PK molecules 43.

Classification of TKIs has gone through an evolution 44-49. Now, according to Roskoski, there are six classes of protein kinase inhibitors (PKIs) with different binding properties 50 (summarized in **Table 1**).

Up to this date, there are 55 small molecule PKIs approved by FDA out of which 39 are TKIs (**Table 2**). Most FDA-approved TKIs fall within Types I to II. An online PKI database, managed by SB&C Team from Institute of Organic and Analytical Chemistry of the University of Orleans, France, compiles all PKIs, even the ones investigated in clinical trials. At present, there are 117 TKIs listed. One in phase 0, seven in phase I, twenty-nine in phase II, thirty-six in phase III and lastly forty-three in phase IV, out of which five were not approved by FDA 51.

The principle of TKI function is based on a phenomenon called “oncogene addiction” 52. In other words, cancer cell's prosperity is dependent on a single pathway. Members of this pathway become drug targets, and their inhibition ideally suppresses neoplastic growth. However, kinase signaling is rather an intricate spider web than a linear pathway, and disruptions are eventually bypassed, causing tumor to regain its proliferation capacity. The higher the selectivity of therapy, the higher is the risk of the tumor to develop an escape scenario. Drug resistance against TKIs usually emerges within one year of therapy 13 and can be caused by various intrinsic and extrinsic factors, like secondary target gene amplification, mutation or alternative splicing, activation of compensatory pathways or loops, structural (phenotype/histotype/epithelial-mesenchymal transition) changes, increased production of growth factors, changes in glucose metabolism or paracrine-autocrine overactivation 53. Nearly all TKI-resistant tumors show oversignaling activity by receptor overexpression or overactivation 54. This leads to development of new drugs targeting resistant variants. However, even to these mutant-specific inhibitors, resistance will inevitably occur 42. Therefore, more systemic approach might lead to better results in cancer therapy. Combination of different types of inhibitors or combination of TKIs with classic chemotherapy or immunotherapy might prove beneficial 13. Another highly promising approach is a conjunction of TKIs with nanoscaled transporters, which could lead to elimination of adverse effects of treatment on healthy tissue, known for TKI-based therapy (disorders concerning nerves, eyes, heart, lungs, liver, gastrointestinal tract, kidneys, muscles, bones, circulatory and lymphatic system, skin and reproductive system) 54. Beyond the adverse effects, nanomedicine has much more to offer in order to improve the outcomes of patients treated with TKIs. These phenomena are discussed in following chapters.

## Nanomedicine

The main objective for nanomedicine is to secure the distribution of the drug to the desired target - tumor cells - in an adequate local concentration with minimal loss of their volume or activity in the blood circulation. Moreover, it is able to reduce the potential toxicity towards healthy cells and tissues, as well as to avoid acquired drug resistance 55. Wide range of nanomaterials based on metals, non-metals, polymers and liposomes is available, and each of the delivery systems has their specific advantages and disadvantages stemming from their biocompatibility, stability, capacity of loading and release and costs 56, 57.

Composition of nanoparticles (NPs), as well as size, morphology and surface charge can have influence on their biological effect and distribution in the organism. NPs larger than 150 nm accumulate mostly in lungs, liver and spleen, but avoid bioaccumulation and clearance by kidneys. Small NPs (under 5 nm) behave in the opposite manner. NPs in between are less likely to be caught in lungs than in liver and spleen 58. Spherically-shaped NPs are mostly captured in liver, whereas rod-like NPs stay in liver and spleen 59. Extravasation of NPs out of the blood vessels is easier for discoidal-shaped NPs because they rarely exhibit laminar flow, and therefore have more chances to get into proximity with receptors on vascular endothelial cells or to move into adjacent capillaries. On the other hand, internalization of NPs into the cell is easier with smaller cell-NP contact points; therefore, spherical and ovoidal NPs will internalize more easily than elongated NPs that lie flat on the cell surface 60. NPs with positive surface charge are more likely to get filtered out of bloodstream by lungs, liver and spleen, and are therefore least suitable for maintaining prolonged blood circulation 61. Importantly, properties of NPs can be improved by various modifications affecting surface charge, providing functional groups or coating for attachment of ligands, such as antibodies, proteins, and peptides, to enhance drug bioavailability, prolong blood circulation or facilitate active targeting 62-67.

In the fashion of poorly soluble small molecule anticancer drugs 68, 69, TKIs are recently in the focus of nanomedicine research. Recent review written by Moradpour and Barghi discusses different types of targeted delivery of TKIs 70. In contrast to this paper, we focus on the particulars of nanoconstruct applications in combination with TKIs. As a brief overview of the applicability of nanoscaled materials in conjunction with several types of TKIs, **Table 3** summarizes recent reports concerning this topic.

### Nanomaterials-based improvement of pharmacokinetics of TKIs

**Figure 2:**
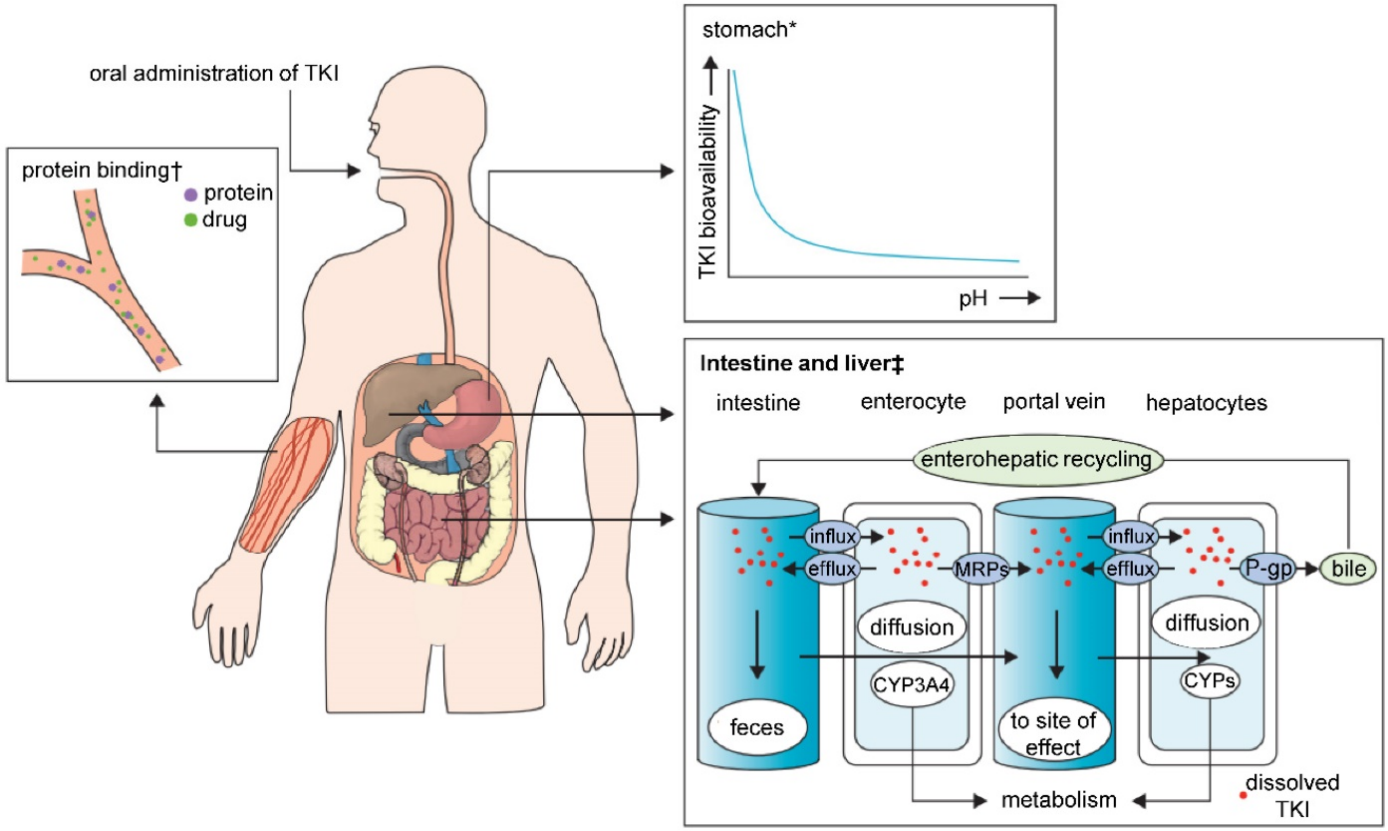
Major sites of absorption, pharmacokinetic drug-drug interactions and elimination in TKI therapy. Abbreviations: TKI, tyrosine kinase inhibitor; MRPs, multidrug resistance protein drug transporters; P-gp, P-glycoprotein; CYPs, cytochrome P450 enzymes. *With increasing stomach pH the bioavailability of TKIs decreases. †Protein-bound molecules are not available to exert pharmacological effects. ‡Shows the association between CYPs and drug transporters in TKI absorption or metabolism. Adapted from Van Leeuwen *et al*. 71 with publisher´s permission (license no. 4802950894525).

TKIs are characterized by poor solubility, and thus highly variable bioavailability manifesting through diverse characteristics regarding absorption from the gastrointestinal tract (**Figure 2**). Major determinant in TKI absorption is the pH-dependent solubility. TKIs exhibit weakly basic properties; therefore, the luminal pH of the gastrointestinal tract and the pK_a_ of the drug determine whether they take the ionized or non‑ionized form. In the highly acidic environment of the stomach, the equilibrium is inclined to the ionized form, which normally dissolves more easily than a non‑ionized form. Bioavailability can be impaired by co‑administration of TKIs with an acid-suppressive medication [e.g. antacids, proton pump inhibitors (PPI)], which increases the stomach pH. The balance of both forms of the drug would shift to the less soluble, non-ionized one and may result in a significant subtherapeutic exposure and treatment failure 71-73. TKIs that are lipophilic and non-polarized at physiological pH dissolve freely in lipids, and therefore easily diffuse passively through the lipid bilayer membrane. Polarized TKIs, on the other hand, are commonly transported by proteins that form transmembrane channels 74, 75. These properties of TKIs pose a major challenge for their use in clinical practice, and it has to be noted that nanomedicine is highly promising to solve these issues. Through encapsulation or complexation of TKIs, nanomedicines are capable of: *i*) protecting TKIs from the harsh conditions of gastrointestinal tract, *ii*) improving the intestinal absorption of TKIs and *iii*) provide TKIs with a controlled release and sustained ionization rates. Interestingly, despite the fact that most of the TKIs get manufactured for the oral administration, nearly all of the FDA-approved nanomedicines are developed for i.v. applications. This implicates an essential need for more advanced nanovehicles able to cope with the obstacles for orally-administered nanomedicines.

When administered i.v., all TKIs are highly bound (most of them over 90%) to plasma proteins (most often to α(1)-acid glycoprotein and/or albumin), which makes these inhibitors ineffective 76. TKIs are also characterized by relatively long plasma half‑life, ranging from shorter time spans, like dasatinib (3-5 h), to long ones, like sunitinib (40-60 h). In this unilateral point of view, the drug stability in the bloodstream should be sufficient to reach desired target without the help of nanoformulation. However, short term maximum tolerable dose of TKIs can be transformed into intolerable toxic dose with chronic long-term drug exposure. Another reason to consider nanoformulation could be the low bioavailability of TKIs that is affected by their hydrophobicity, even though there are exceptions (e.g. imatinib, erlotinib) 54.

Nowadays, mostly rat and mouse models are used for *in vivo* studies of TKI nanoformulations. Therefore, we cannot compare actual data from clinical usage of TKIs with pharmacokinetic abilities of nanoformulated drugs tested on animals. However, recent studies showed that nanoformulations can markedly improve TKIs pharmacokinetic properties in *in vivo* settings.

For example, lipid-based nanoformulations, being amphipathic, are able to enhance solubility and bioavailability of TKIs. This phenomenon was demonstrated by Dora *et al*., who constructed a phospholipid complex with erlotinib. Pharmacokinetic assessment showed ∼1.7-fold (*p* < 0.05) higher bioavailability of the nanoformulated complex compared to free drug, with ∼1.3-fold (*p* < 0.01) higher maximum plasma concentration and longer half-life. 77. The profound pharmacokinetic improvement of TKIs can be explained by the enhancement of their hydrophilicity achieved through complexation with phospholipid complexes together with prolonged exposure to the molecular complex due to higher mean-residence time in a proximity of tumor tissue. Similarly, Qiu *et al*. prepared a phospholipid complex with ibrutinib, which exhibited 9.14-fold increase in bioavailability compared to free drug. Maximum plasma concentration was also higher and was achieved in shorter time period, while tumor volume was significantly decreased 78.

Nazari-Vanani *et al*. used sunitinib maleate in combination with self-nanoemulsifying delivery system (SNEDDS) that is characterized by a high hydrophilic-lipophilic balance. In an *in vivo* experiment, oral administration of nanoformulation had higher plasma concentration over time, with 1.45 times higher maximum concentration achieved in less than half the time 79. In another recently published study, Lu *et al.* constructed liposomal afatinib with cetuximab (anti-EGFR antibody) coating. These immuno-NPs showed tumor growth inhibition and higher afatinib concentration in tumor tissue, as well as in liver and lungs. Due to the prolonged blood circulation and inhibited binding rate to endogenous proteins, afatinib nanoformulation exhibited higher area under curve (AUC) and plasma terminal half-life, when it could be detected even after 72 h in the bloodstream 80.

Gao *et al*. utilized human serum albumin (HSA), which, as the main protein for plasma transport in physiological conditions, can escape systemic clearance naturally and has higher uptake in cancer cells, because they are in need of nutrients for their uncontrolled growth 81. Aware of these advantages, they prepared NPs coated with sorafenib and with or without grafted folic acid (FA), and studied their effect on hepatocellular carcinoma. Nanoformulation increased AUC, mean residence time and plasma half-life and decreased total plasma clearance, which is a positive result. NPs without grafted FA showed better values than the ones with FA. However, the latter ones markedly increased sorafenib accumulation in tumor tissue and reduced the tumor volume 82.

Li *et al*. produced biomimetic galactose-modified polymeric NPs loaded with imatinib that exhibited enhanced oral bioavailability, higher maximum concentration and AUC with increased intestinal permeability and affinity to intestinal epithelial cells. Fast entrance into the bloodstream resulted in fast clearance in liver, and NPs were not able to extend plasma residence time. Authors suggest NP modification with functional biomaterials to maintain imatinib plasma concentration for longer period. However, benefit of this nanoformulation lies in fast bloodstream penetration, as it might result in adverse events reduction 83.

Taken together, inherent physico-chemical properties of TKIs are substantially complicating their treatment efficiency. Abovementioned studies clearly demonstrate that distinct types of nanomedicines can markedly improve the pharmacokinetic behavior of TKIs. This in particular is due to improved solubility of TKIs incorporated into delivery vehicles, followed by a subsequent enhancement of TKIs hydrophilicity and improved stability in the blood stream that results in a prolonged blood circulation of nanoformulations and their higher residence time in tissues adjacent to tumor mass. Even passive, non-targeted nanoformulations can be then accumulated in the tumor mass through “enhanced permeability and retention” (EPR) effect 84. Additional fine-tuning of nanovehicles (*e.g.* involvement of ligands mediating active targeting of specific subsets of cancer cells or utilization of tumor microenvironment stimuli-responsive domains) could further improve the pharmacokinetics of TKI nanoformulations.

### Reduction of adverse effects of TKIs-based therapy

Although targeted therapy is, in general, regarded as more moderate than classic chemotherapy, it has more intricate mechanisms of action concerning their structure and targets. Even in targeted therapy, severe adverse events occur and resulting dose adjustments or treatment discontinuation impair its better treatment efficacy 85.

Eckstein *et al*. summarized toxicity characteristics of eleven TKI drugs available on the market. They pooled the side effects classification scale into two groups, the first including rare and uncommon events, the second involving common and very common events. All eleven inhibitors caused nerve, lung and airways disorders with the higher abundance. Also, in the same abundance category, ten caused skin disorders, nine caused gastrointestinal disorders, carcinogenic, mutagenic and toxic effects on reproductive system, and eight of them caused eye, blood and lymphatic disorders. Other adverse effects were related to liver, bile, vascular system, kidneys or musculoskeletal system. Pazopanib, as the most severe inhibitor from the list, had 12 out of 14 indications with the frequency of common, very common. On the other hand, sorafenib had only 5 common or very common effects out of 14 54.

As demonstrated in the previous paragraph, pooling of adverse events grading quite commonly occurs in studies and articles. This bad habit may distort the actual graveness of some events. For example, in Common terminology criteria for adverse events (CTCAE v5.0), grade 1 plus grade 2 and grade 3 plus grade 4 events are regularly pooled, where main focus goes to the grade 3/4 group. However, in the case of long-term drug administration, group 1/2 should not be overlooked, if we want to improve patient's quality of life. It is important to keep in mind that the milder but more common side effects put a significant impairment on patient's everyday psyche 85.

In this sense, well-thought-out design in drug development, substantial drug toxicity profiling and proper administration are needed to minimize adverse events. Nanoformulations have good potential to reduce these events even more by specific biodistribution of anti-cancer drugs. In preclinical *in vitro* and *in vivo* models, number of papers provide evidence that drug-loaded NPs show enhanced properties compared to free drugs. For example, Cryer *et al*. developed gold NPs conjugated with an afatinib analog that mediated cancer cell-specific growth inhibition and attenuated inflammatory cytokine release. The selectivity to cancer cells is plausibly due to highly efficient uptake of gold NPs concomitantly inhibiting the efflux of afatinib to the extracellular space 86. Marslin *et al.* evaluated performance of imatinib mesylate-loaded poly(lactide-co-glycolide) NPs that exhibited increased cytotoxicity *in vitro* and significantly reduced cardiotoxicity *in vivo* compared to free drug. 87. Despite the fact that the authors did not investigate phenomenon responsible for inhibition of imatinib side effects, it is likely that loading of imatinib into NPs results in improved blood circulation and preferential bioaccumulation of imatinib in tumor mass due to the EPR effect. Wan *et al.* worked with lapatinib-loaded HSA NPs on HER2 positive 88 and triple negative metastatic breast cancer 89. The former, apart from showing enhanced anti-tumor activity, manifested no subchronic toxicity throughout the duration of the experiment. The latter one improved inhibition of adhesion, invasion and migration of brain-metastatic breast cancer cells. *In vivo*, the nanoformulation increased delivery to brain tissue and significantly prolonged median survival period of tested mice.

The relative selectivity of nanodrugs in cancer is based on the so-called passive targeting by the abovementioned EPR effect. This mechanism is based on substantial extravasation of the nanodrugs into the interstitial fluid at the tumor site. Nanodrugs can better penetrate the tumor due to poor quality vessels in the tumor and are retained for a longer time at the tumor site because of the defective lymphatic vessel system in tumor microenvironment. EPR affects nanodrugs that are 20-100 nm in size 90. Improved therapeutic effects of drug nanocarriers can be achieved by targeting molecules specific to a particular tissue or cell type. Song *et al*. prepared cyclic arginylglycylaspartic acid (cRGD) and polyethylene glycol (PEG) modified liposomes with apatinib that specifically recognize integrin αvβ3 91. In comparison with free drug and untargeted liposomes, the cRGD-Lipo-PEG/Apa showed enhanced cytotoxicity and cellular uptake *in vitro*, plus it performed better targeting, faster clearance from murine organism, significantly reduced tumor weight and no visible damage to internal organs. Lu *et al*. intended to overcome adverse effects of combination therapy with afatinib and cetuximab by encapsulating the TKI into liposomes and using cetuximab as targeting and therapeutic agents bound to the surface of liposomes 80. This solution resulted in better cellular uptake and *in vivo* prolonged half-life, better drug delivery, prevention of afatinib binding to hemoglobin, enhanced tumor selectivity and growth inhibition. This study suggested the drug dosage could be significantly decreased in clinical application, therefore potentially reducing adverse events incidence.

### Blood-brain barrier

A number of targets have been identified for potential treatment of brain malignancies by TKIs (e. g. EGFR, VEGFR, AKT, ALK, c-Met) 92. At least 50% of brain metastases are caused by lung cancer. Second most common (15-25%) metastasis originates in breast cancer tissue 93. Both of their non-metastatic precursors are commonly treated with TKIs. However, major problem in brain cancer therapy is connected to the blood-brain barrier (BBB) - the specific lining of brain blood vessels characterized by intercellular adherens and paracellular tight junctions, specific efflux transporters for nutrients (solute carriers) and metabolites (ATP binding cassette) and absence of intracellular vesicular transport. Nature of the BBB helps protect the brain tissue from passive diffusion of molecules, mainly to avoid toxins and harmful substances 94. Although the BBB gets disrupted by tumor to better supply nutrients, this only happens in the center of the tumor mass. In the peripheral areas, blood-brain tumor barrier exists, and it is similar to BBB 95. All of this, unfortunately, works against brain disease therapy, and many small-molecule inhibitors did not find success in reaching their target behind the BBB 92.

**Figure 3:**
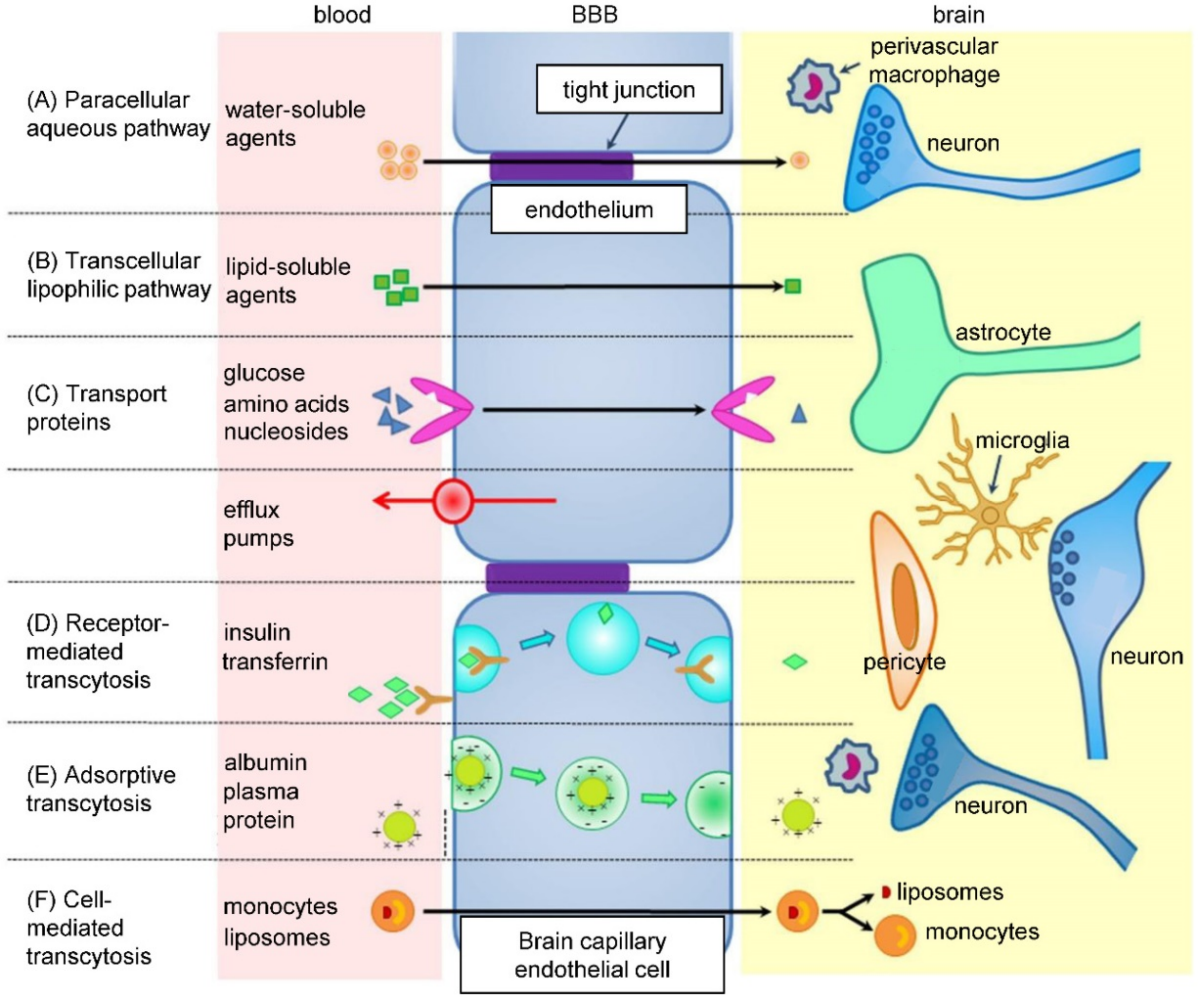
Pathways across the BBB. BBB consists of endothelial cells linked by tight junctions, encircled by pericytes and astrocytes, together with basal lamina and neurons. In between adjacent cells (paracellular pathway - A), some ions and solutes cross freely by passive diffusion. Other molecules utilize various transcellular pathways (B-F), like specific transporter proteins or receptor-mediated transcytosis and diffusion. Efflux transporters, such as P-glycoprotein (P-gp), prevent some substances from crossing the BBB. Adapted from Wang and Wu 206 with publisher´s permission (license no. 4892530338842).

Nanomedicine gives drugs the advantage of prolonged blood circulation time, which increases their chance to cross the BBB (**Figure 3**) 96. Alternatively, NPs can increase drug concentration in the proximity of BBB cells, which could cause a local disruption of the BBB and allow the drug to penetrate inside the brain 97. More importantly, NPs can be designed to possess the ability to cross the BBB themselves and successfully deliver the drug. For example, surface charge of NPs has an effect on the BBB - neutral or lower concentrations of anionic NPs are suitable for brain penetration, as opposed to cationic NPs, which exhibit toxicity to brain endothelium 98. Targeting ligand, such as transferrin, insulin, glutathione, HIV-1 trans-activating transcriptor or peptide derived from apolipoprotein-E, can be attached to the surface of NPs to ensure crossing the BBB by receptor-mediated transcytosis 99. However, after overcoming the BBB, NPs have to be able to internalize into tumor cells as well 96.

Wan *et al*. prepared HSA NPs for treatment of triple negative breast cancer brain metastases with lapatinib 89. *In vitro* analysis showed that, compared to free drug, the nanoconstruct suppressed the adhesion, migration and invasion ability of 4T1 cell line with better efficacy. *In vivo*, lapatinib concentration in the brain of 4T1 injected mice was the highest in NPs-treated group at all time points after administration. High brain tissue accumulation of lapatinib was plausibly due to capability of HSA NPs to inhibit the efflux of the drug by P-gp and BCRP transporters, restricting the retention of free lapatinib in the brain. In a survival experiment, brain metastatic nude mice were treated with three different concentrations of nanoformulated lapatinib, as well as free drug solution and commercially available drug Tykerb. Each nanoformulation prolonged mice survival compared to non-nano formulations, while the highest concentration (30 mg/kg) resulted in the longest survival time.

To improve the transcytosis of lapatinib across the BBB, Wan *et al*. relied on enhanced EPR effect plus attraction of HSA NPs by SPARC (secreted protein acidic and rich in cysteine), as well as on beneficial properties of HSA that include preferential tumor uptake and binding to 60-kDa glycoprotein receptor on vascular endothelial cells 89. Lakkadwala *et al*. took up different approach - liposomes modified with transferrin and penetratin loaded with erlotinib and doxorubicin for treatment of glioblastoma 100. Surface functionalization mediated intracellular uptake, which was demonstrated in this study. Erlotinib and doxorubicin concentration in brain tissue was significantly higher using dual functionalized nanocarriers than free drugs or single peptide-modified liposomes, suggesting utmost importance of receptor-mediated endocytosis (transferrin) and enhanced cell penetration (penetratin) for efficient translocation of erlotinib-loaded liposomes across the BBB. Importantly, survival of glioblastoma bearing nude mice was significantly prolonged and tumor area was minimized.

Although research in brain-targeted nanomedicine is in full bloom, not many researchers focus on using nanoformulations for TKI treatment of brain cancer or metastases. However, these few promising examples show that TKIs could be used in this manner. We postulate that future investigations might focus on development of highly biocompatible nanovehicles of endogenous origin, able to utilize the inherent properties of BBB to mediate transcytosis of nanoformulations. In addition, nanovehicles for TKIs brain delivery might also be capable of inhibiting frequent brain-associated efflux mechanisms causing undesired transport of the payload to the extracellular region.

## Nano-based combination therapy with TKIs

Therapy that combines two or more anticancer agents is applied for two main reasons - to bypass/delay drug resistance or to enhance drug efficacy. Moreover, combination of drugs usually allows reducing the doses of individual drugs, and thus alleviates side effects. Main strategy would be to co-target compensatory signaling pathways with either TKIs or monoclonal antibodies. Some strategies are focusing on Heat shock protein 90 that is necessary for protein stabilization and plays a role in the folding of many RTKs 101-103. Another possibility is to combine TKIs with immune therapy for better tumor recognition by the immune system 53. TKIs can be also used in combination with standard chemotherapy or radiotherapy 2. However, recent research brought to attention novel combinations of TKIs with diverse spectrum of other agents, for example curcumin 104, oxygen 105, chloroquine 106 or even miRNA for regulation of expression of cancer-associated genes 107. In addition, a number of clinical trials studying TKI combination therapy are currently underway or recruiting participants. Studies frequently focus on TKI usage with immunotherapy (NCT04055792, NCT04044378, NCT04042116, and NCT03580928) and chemotherapy (NCT04035486, NCT01931098, NCT00390793, NCT00557193). Combination with different TKIs (NCT04028778, NCT01531361, NCT01982955, NCT02824458 and NCT02767804) is also very common. Fewer trials investigate applications of TKIs with hormonal therapy (NCT04033172) or other agents (PPI - NCT02800330, dendritic cell vaccines - NCT01876212, hydroxychloroquine - NCT00813423, various inhibitors - NCT00335764, NCT00655655, eicosapentaenoic acid - NCT04006847, etc.). Several trials explore combination therapy of TKIs and nanoformulation of other drugs - most frequently it is nanoformulated paclitaxel (e.g. NCT01455389, NCT00733408, NCT00331630, NCT00709761, NCT03942068 and NCT00313599). Importantly, no downright nano-TKI applications reached the stage of clinical research yet.

Multidrug approach has been previously found beneficial in the control of HIV, showing that specific drug combinations were able to overcome treatment difficulties 85. However, the approach to TKI combination therapy should not be global. Tumor/patient specific approach is a prerequisite for making a diagnostic decision whether to use one multi-targeted TKI or several single-targeted inhibitors. Multidrug therapy is generally more suitable for tumors with higher number of overexpressed kinases responsible for resistance and for tumors with resistance induced by a mutation in the TK. Combination TKI therapy requires knowledge of toxicity profiles and metabolism routes for each drug. Tumor microenvironment is also quite important, because gene expression of its stromal cells may be different from the neoplastic cells. Therefore the effect of each TKI on tumor stroma might also be different 108.

Use of drug combination in nanoformulation should be well devised, as there are some drugs that properly function only in specific order and timeframe. Their simultaneous release would cause severe side effects 109.

### TKIs in combination with other small molecule drugs

Combination therapy of two different TKIs has its benefits in targeting multiple TK signaling pathways, generating more potent anti-tumor effects and limiting possibilities of bypassing the inhibited pathway, thus avoiding or postponing the occurrence of resistance. Archibald *et al.* showed this on castration-resistant prostate cancer cell lines with the combination of sorafenib and nilotinib 110. Greish *et al.* proved that crizotinib and dasatinib combination in micellar formulation shows enhanced anti-tumor efficacy against glioblastoma multiforme, possibly due to increased blood circulation and improved bioaccumulation of TKIs at the tumor site 111.

There are records of using chloroquine in combination with nanoformulated TKIs based on its multiple mechanisms of action affecting tumor microenvironment. Chloroquine can inhibit late stage autophagy, activate tumor suppressor protein p53, normalize tumor vasculature, which decreases hypoxia, *etc*. 112. In this way, Zhao* et al.* delivered chloroquine/gefitinib cocktail encapsulated in chitosan NPs to resistant hepatocellular carcinoma cells and managed to overcome said resistance through inhibition of the autophagic effects, evidenced by down-regulation of the LC3 II and LC3 I ratio 113. Similarly, Lv *et al.* made polyamidoamine NPs functionalized with anti-EGFR aptamers for targeted co-delivery of erlotinib and survivin-shRNA with simultaneous systemic administration of chloroquine. Purpose of chloroquine in this case was mainly vascular normalization for smooth NPs delivery and endosomal escape, while therapeutic effects were mediated by erlotinib and shRNA down-regulation of survivin that inhibits drug-induced apoptosis 114.

Interestingly, Hu *et al*. developed a thermosensitive hydrogel for peritumoral use, containing a nanoformulated Pluronic F127-based chemotherapeutic agent paclitaxel with short release time and lapatinib microparticles with long-term release kinetics 115. Initial high concentration of paclitaxel strongly suppressed tumor growth, and the stable lapatinib release maintained its efficiency to enhance the therapeutic impact. *In vivo* experiment showed that in comparison with oral administration of lapatinib, the gel administration performed comparably, although with much smaller amount of drug needed. In addition, by using the gel, steadier bioaccumulation of lapatinib in tumors together with lower undesired toxicity was achieved, and nanoformulated lapatinib exhibited good synergistic effects with paclitaxel.

### TKIs in combination with immunotherapy

In some cases, antibodies are used in combination with TKIs in nanoconstructs to target TK domains for precise delivery and for their anti-tumor activity in the form of competitive ligand binding 116, 117. Other times, TKIs like sunitinib are used in combination with immunotherapy for their ability to modulate tumor microenvironment 118. For example, Huo *et al.* used sunitinib encapsulated in polymeric (anisamide-modified poly-lactic-glycolic-acid-PEG) micellar nanovehicle to support their tyrosinase-related protein 2 (Trp2) nanovaccine in melanoma therapy, with results showing attenuated tumor-associated immune suppression 119. Polymeric nanovehicles enabled an efficient delivery of a low dose of sunitinib and decreased the number of myeloid-derived suppressor and regulatory T cells with enhanced infiltration of cytotoxic T cells, thereby abrogating tumor-associated immune suppression. Kim *et al.* combined their Toll-like receptor 7/8 agonist nanovaccine with free sunitinib and programmed death-ligand 1 (PD-L1) antibody, which, again, abrogated tumor-associated immune suppression and significantly decreased tumor weight in an *in vivo* experiment 120. Yin *et al.* also used PD-L1; however, they produced a nanobody acting as a targeting ligand of liposomal delivery system of gefitinib and simvastatin. Their goal, to overcome gefitinib resistance in T790M EGFR-mutated NSCLC, was achieved in an *in vivo* experiment 121. The studies have clearly demonstrated that nanoformulations of TKIs could be useful as synergistic modalities to improve the outcomes of existing immunotherapies.

### TKIs in combination with other biologically active agents

There have been studies combining TKIs with natural substances that have multiple effects on human metabolism. For example, Zhao *et al*. combined sorafenib with ursolic acid in mesoporous silica NPs coated with pH sensitive chitosan and lactobionic acid - targeting agent for asialoglycoprotein receptor overexpressed in hepatocellular carcinoma. They used ursolic acid for its reported ability to down-regulate VEGFR and EGFR 122. Ursolic acid is said to have profound anticancer properties 123. Zhao's data hint a synergy of both free drugs combined; however, NPs showed the best results in anti-tumor activity, particularly due to their ability to trigger the release of payloads in response to acidic pH of tumor microenvironment.

Chen *et al.* combined sunitinib with curcumin in superparamagnetic iron oxide NPs coated with BSA 104. Curcumin was found to have an anti-invasion and anti-metastatic effect 124, 125 and is quite popular in nanomedicine nowadays 126-131. In Chen's study, nanoformulation showed markedly better results than free drugs alone or in combination. This was achieved through the capability of the vehicle to efficiently deliver the payload and to strictly maintain the optimal ratio of sunitinib and curcumin at the tumor mass toward the most optimal synergistic cytotoxic effect 104. Cao *et al.* used sorafenib/curcumin cocktail encapsulated in directed self-assembled nanoparticles composed of PEG derivative of vitamin E succinate with similar results. Upon oral administration, significantly increased biodistribution of sorafenib and curcumin in gastrointestinal tract was found. The authors stated that this phenomenon is most likely due to the improvement of the affinity and accessibility of nanoformulations to the intestinal membrane that results in their enhanced absorption in the intestine 132.

Hypoxia occurs in the center mass of solid tumors. It stimulates overexpression of HIF-1α that mediates signaling pathways leading to epithelial-mesenchymal transition, neovascularization, cell survival, metastasis and therapy resistance 133. In NSCLC with mutated and wild-type EGFR subjected to hypoxia, significantly upregulated HIF-1α and TGFα were found, and resistance to gefitinib treatment occurred 134. Li *et al.* co-delivered oxygen-releasing perfluorooctylbromide with erlotinib encapsulated in anti-EGFR aptamer-modified liposomal NPs to overcome hypoxia-induced resistance to chemotherapy. NPs with combination of drugs had significant reducing effect on tumor volume compared to NPs with erlotinib alone, which suggests good therapeutic synergy. The study highlights the importance of active targeting moiety on the surface of nanoformulation. Anti-EGFR aptamer enables specific ligand-receptor mediated accumulation of the payload enhancing efficiency of encapsulated drugs with concomitant inhibition of undesired side-effects *in vivo* 105.

## Theranostics

Theranostic approach unites within itself the therapeutic and diagnostic aspects of medicine. In another words, it is the combination of therapy and pharmacokinetic imaging *in situ* that enables long-term monitoring, and therefore adaptation to modality of disease prognosis. This approach addresses pharmacokinetic variability of drug responses in the population of patients, hopefully resulting in a reduction in adverse event occurrence and in an improvement of treatment planning 135. Nanotechnology became a suitable theranostic tool, bringing in its benefits, like the ability to carry a single drug or drug combination, controlled and sustained release of its load, ability to bind targeting moiety for specific delivery, *etc*. 136.

**Figure 4:**
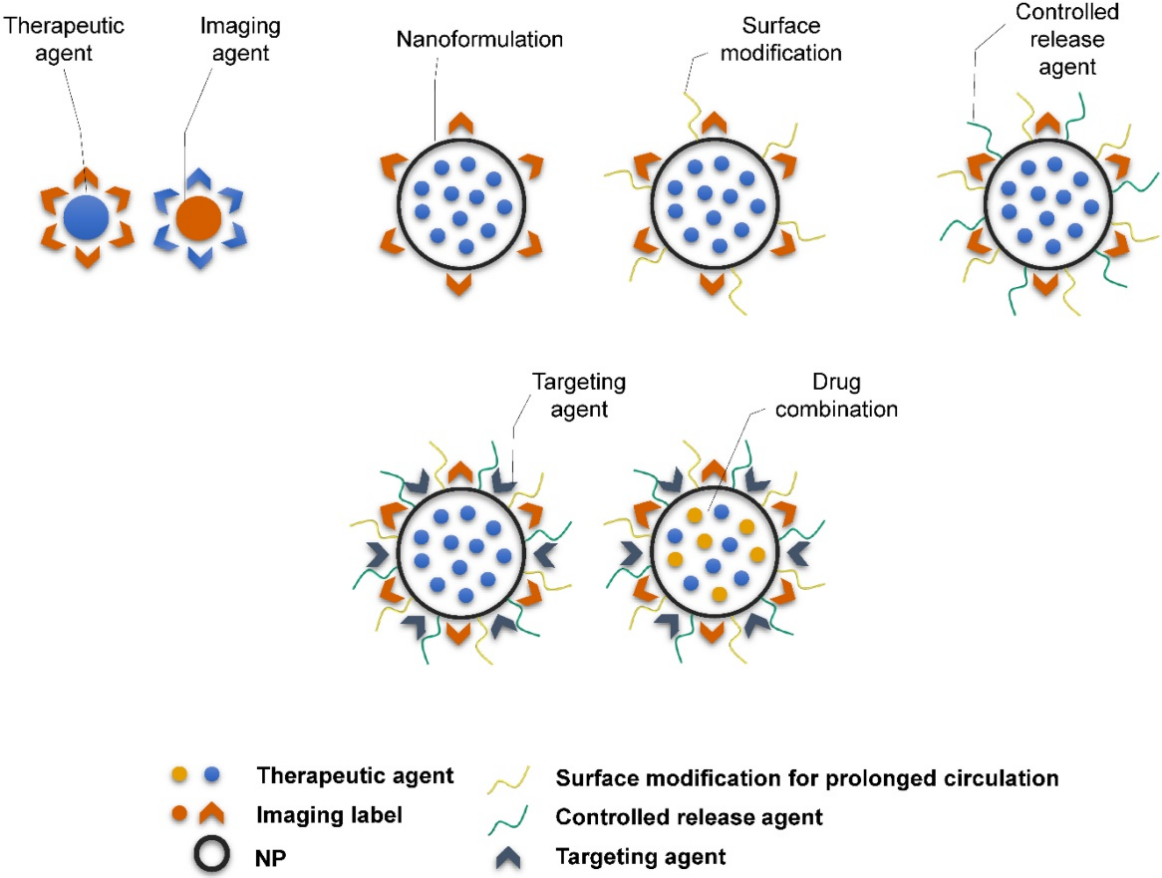
Schematic representation of the complexity of theranostic agents'. Basic approach employs a therapeutic molecule conjugated with a label. Nanoformulation allows attaching variety of different ligands and functional surface modifications to achieve prolonged blood circulation (e.g. PEGylation), controlled release of therapeutic agent (e.g. chitosan) or targeting for particular tissue or cell type (e.g. antibodies, peptides).

**Figure 5:**
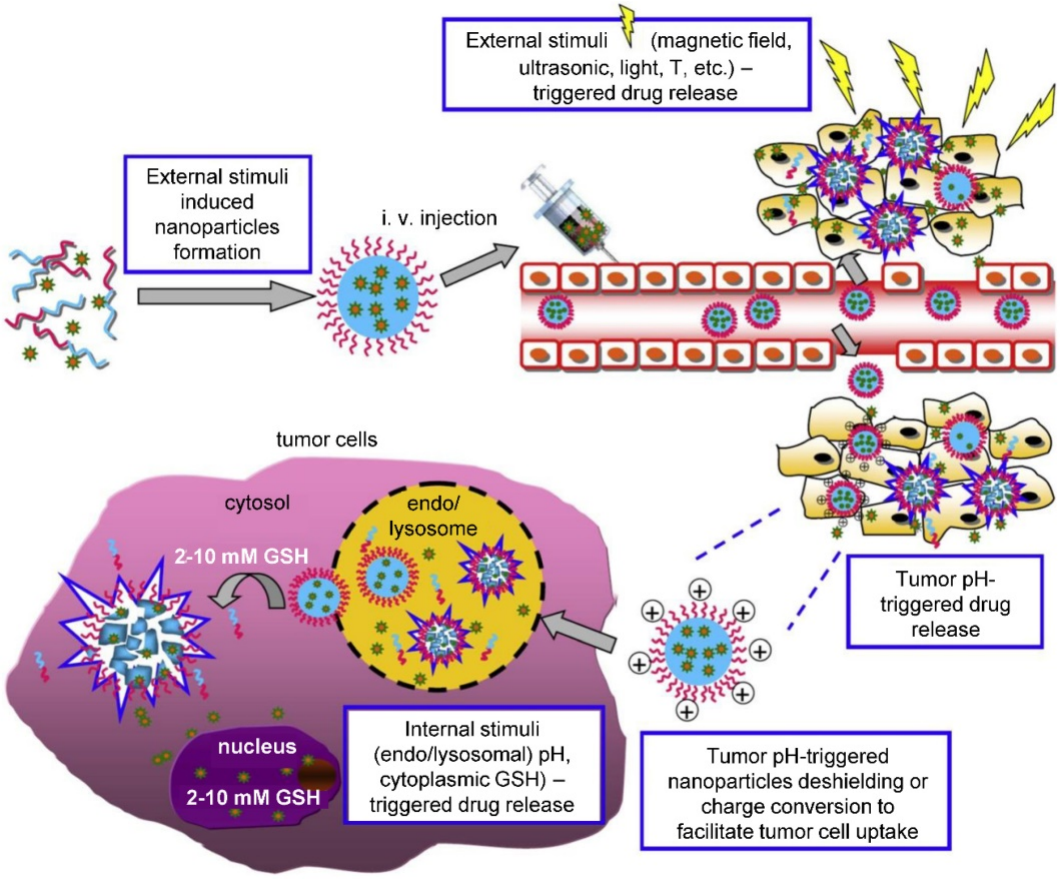
External and internal stimuli for controlled drug release. External stimuli allow for time and site-specific drug release triggered by the application of physical forces, as is magnetic field, temperature, ultrasound, electromagnetic field, light etc. On the other hand, internal stimuli stem from the nature of the tumor and its microenvironment, where specific pH, redox potential or enzyme activity act as a trigger in the tumor tissue or intracellularly. Adapted from Cheng *et al*. 139 with publisher´s permission (license no. 4892530661903).

Theranostic agents typically consist of a therapeutic molecule and a traceable label (**Figure 4**). In case of using nanocarriers, targeting agent can be added, or solely the EPR effect of vehicles in the tumor tissue can be utilized. Targeting agents are typically small molecules, peptides, proteins or antibodies. The variety of labels used in theranostics corresponds to the selection of imaging techniques. For instance, gamma scintigraphy (positron emission or single-photon emission computed tomography) is suitable for visualization of radionuclides (e.g. ^18^F, ^111^In, ^64^Cu). Optical methods commonly detect fluorescent labels, quantum dots, carbon nanotubes or gold nanoshells. Magnetic resonance imaging (MRI) is a good method for para- or superparamagnetic metals, like gadolinium, iron and manganese or NPs from respective materials. Heavy elements, such as iodine, barium and sulphate are visualized by computed tomography, and last but not least, ultrasound can be used to view liposomes and perfluorocarbon nanodroplets 137, 138. Drug release can be controlled by one or more stimuli (**Figure 5**). Some approaches rely on internal cellular stimuli, like pH, redox potential or enzymes. Others trigger drug release with external stimuli, temperature, light, ultrasound and magnetic field. The stimuli combination concept boosts tumor cellular uptake and intracellular drug release while decreasing nonspecific cytotoxicity thanks to improved nanocarrier stability 139.

**Figure 6:**
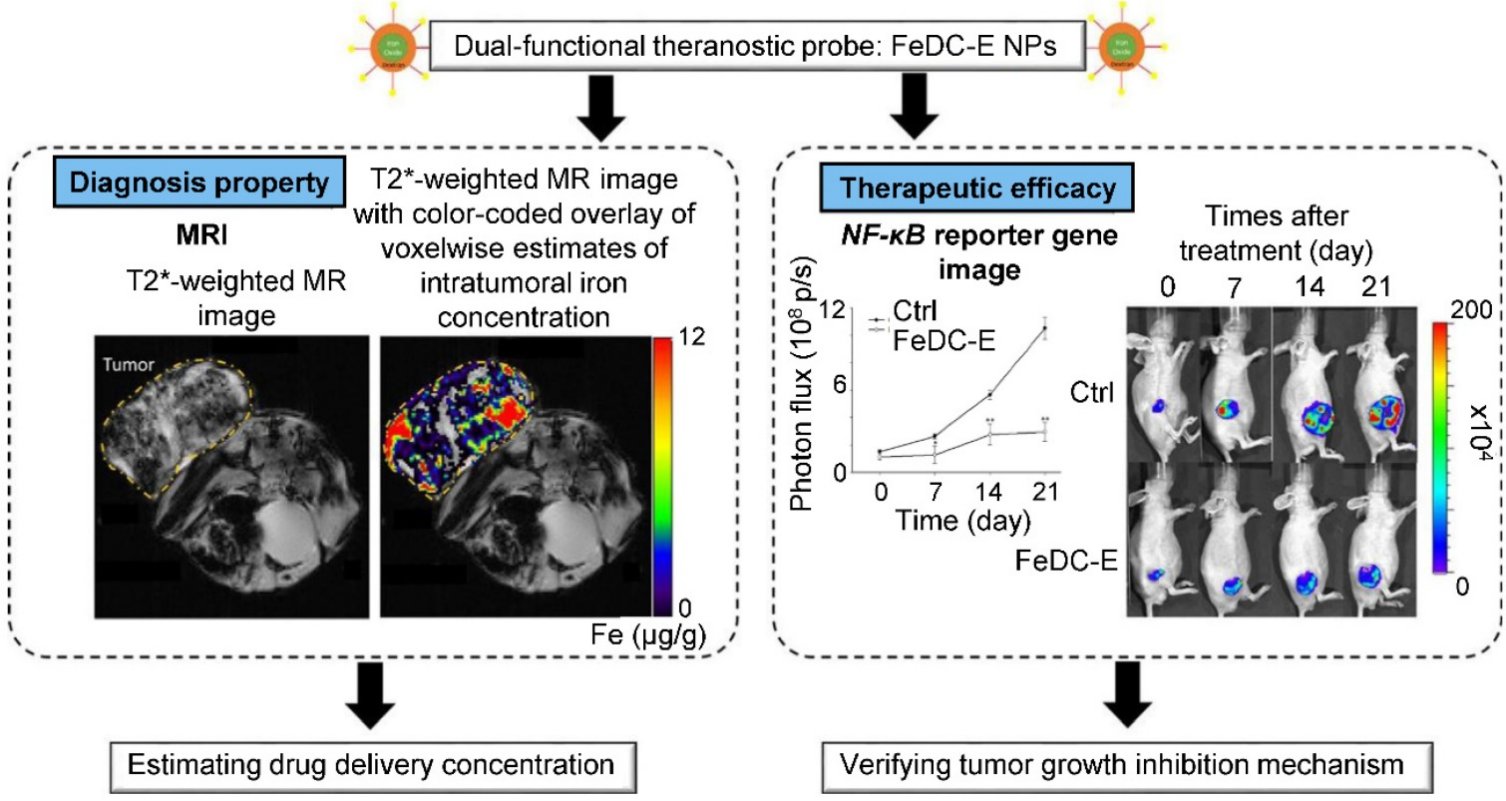
Representation of diagnostic and therapeutic properties of erlotinib-conjugated iron oxide NPs (FeDC-E NPs). Additionally to FeDC-E NPs capability of tumor growth inhibition, demonstrated by decrease of ectopic lung tumor mass and suppression of NF-κB expression captured (*right*), assessment of delivery efficacy and drug concentration estimation can be made, using MRI (*left*). Abbreviations: CTRL, control; FeDC-E NPs, erlotinib-conjugated iron oxide NPs; NF-κB, nuclear factor kappa light-chain enhancer of activated B cells; T2* decay of transverse magnetization caused by a combination of spin-spin relaxation and magnetic field inhomogeneity. Adapted from Hsu *et al*. 207 with publisher´s permission (license no. 4802960989730).

One of the earliest mentions of nano-TKI application in theranostics was made by Noh *et al*., who developed cross-linked iron oxide NPs conjugated with sunitinib for estimation of expression status of VEGFR and PDGFR by MRI. It has been shown that the iron oxide NPs serve as highly biocompatible nanoplatform with exceptional inherent MRI contrast capabilities, offering facile conjugation of sunitinib 140. Liu *et al.* constructed sorafenib-loaded pH-sensitive multi-block polymer [poly(lactic acid)-poly-(ethylene glycol)-poly(l-lysine)-diethylenetriamine glycol)-biotin] NPs with VEGFR targeting by anti-VEFGR antibody and gadolinium ions as MRI contrast agent. *In vitro*, NPs with anti-VEGFR antibody showed higher cellular uptake than NPs without the antibody. *In vivo*, NPs showed better anti-tumor efficacy than sorafenib alone with prolonged imaging time and signal enhancement compared to commercial contrast agent Magnevist®. In addition, NPs exhibited exceptional biocompatibility and did not alter organ histology. Overall, the devised NPs served as highly promising biocompatible, actively-targeted, multifunctional platform capable of facile dual loading with TKI and imaging agent 141. Hsu *et al*. developed erlotinib delivery monitoring system *via* iron oxide NPs with MRI imaging and demonstrated the ability to estimate intake concentration of NPs into the tumor (**Figure 6**) 142. *In vivo* experiments revealed that the erlotinib-conjugated NPs effectively inhibited progression of lung cancer. *Ex vivo* protein expression analysis revealed efficient suppression of antiapoptotic and activation of apoptotic proteins mediated by NF‑κB signaling.

Zhang *et al*. harnessed the full potential of theranostic approach by fabricating indocyanine green-loaded mesoporous silica NPs lidded with zinc oxide quantum dots and coated with erlotinib-modified chitosan 143. This redox/pH dual-responsive system targeted EGFR-mutated NSCLC and worked synergistically with NIR irradiation, which provided targeted therapeutic effects in the form of reactive oxygen species generation and regulation of EGFR and apoptosis-related protein expression. As a last example of possible conjunction of TKIs and nanomaterials for theranostics, Sang *et al.* constructed a complex nanodelivery system with multiple functions. Chitosan oligosaccharide was conjugated with IR780 cyanine fluorescent label and Black Hole Quencher for visualization of fluorescence triggered by NIR irradiation. This complex then self-assembled, encapsulating sorafenib and superparamagnetic iron oxide NPs that were intended for induction of ferroptosis characterized by a high rate of lipid peroxidation 144. This elaborate structure was capable of causing explosive lipid peroxidation upon irradiation with NIR (> 18-fold compared to non-irradiated structure) and exhibited > 26-fold increase in half-life of sorafenib.

The few examples discussed above clearly demonstrate that TKIs-based chemotherapy might substantially benefit from involvement of nanoparticles with theranostic properties. Such structures could markedly improve the efficiency of anticancer therapy by real-time tracing of TKIs accumulation and continuous visualization of chemotherapy outcomes. Theranostic nanoformulations could also offer a reduction of the dose requirements of administered TKIs, which are frequently characterized by narrow therapeutic indices 145.

## Future outlooks

Nanotechnology offers unique properties in drug development. There has already been several nanomedicines approved by FDA 146, and close to 200 clinical studies with “recruiting”, “enrolling”, “active” or “completed” status, are listed on Clinicaltrials.gov under key words “nanoparticle” and “cancer”. However, nanomedicine development still has a lot of room for improvement. Wu *et al*. summarize challenges in this field in their review 147. If we follow the path of the first FDA approved nanomedicine - Doxil 148, we see an eight-year-long path from the initial idea to clinical trials. There are plenty of studies that reached *in vivo* phase, but we do not see these nanodrugs continue to clinical trials yet. For the advancement from *in vivo* to clinical trials, scale-up in nanodrug manufacturing must be achieved. That requires reproducibility and uniformity in production, drug stability and the possibility to involve automation in the production process, if the trials are successful. We see these requirements and hefty financial investment as the bottleneck of clinical transfer of today's nanomedicine. Especially TKI-nanoapplications are in the beginning phases of development and, although they have great potential, they still have a long way ahead of them.

Harnessing the potential of bioinformatics, artificial intelligence (AI) and deep learning could marginally increase the advancement in nanomedicine 149. Shamay *et al.* demonstrated the strengths of computational methods when they showed precise prediction of NP formulation and properties based on molecular structures of precursor molecules, producing NPs with selective targeting ability and impressive therapeutic impact 150. Nanoinformatics could eliminate or at least alleviate the labor-intensive trial and error-based NP development.

**Figure 7:**
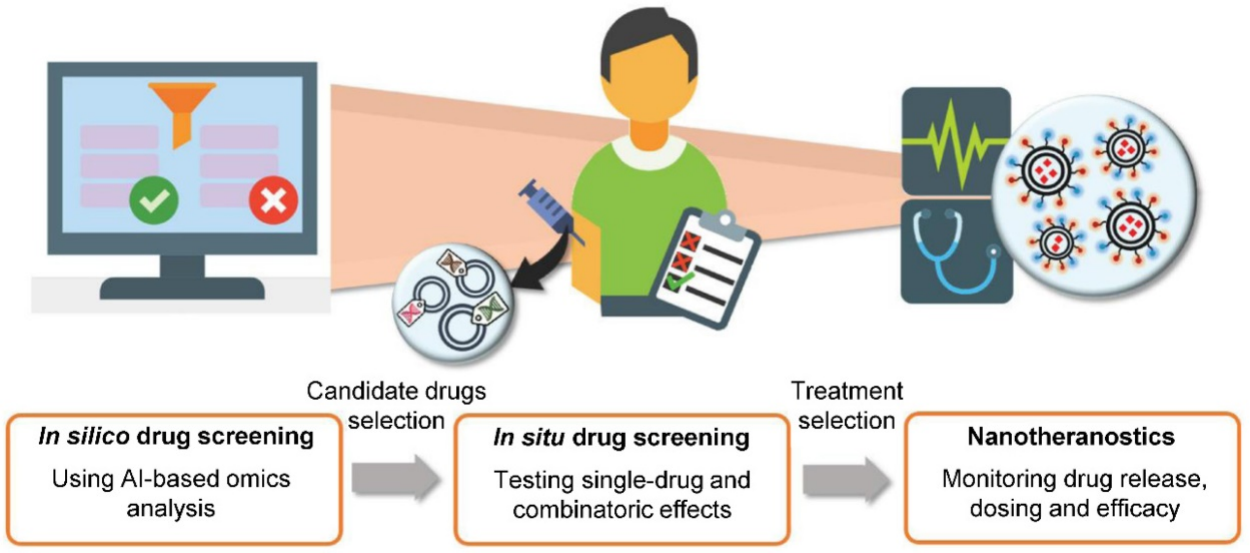
The idea of future personalized medicine. AI-based technologies could be utilized in *de novo* target and drug discovery, prediction of drug efficacy, dosage or combination and response to therapy. Suitable drug candidates and strategies would then be tested and resulting final therapeutic approaches would be monitored by nanotheranostics. Obtained data could be fed back to AI to generate prediction of treatment success and adjustments of treatment plan. Adapted from Adir *et al*. 149 with publisher's permission (license no. 4892531028481).

With growing number of public databases 151 and high-throughput methods, mining for information in large datasets becomes more and more important. The application of machine learning and AI technology introduces broad possibilities, such as drug discovery 152, target discovery 153, 154, predictions of NP properties 150, 155, prediction of phenotypic responses 156, 157, image analysis for clinical diagnosis 158, 159, determination of suitable drug combinations for individualized therapy 160, 161 with subsequent efficacy evaluation and dose adjustments 162 and so on 163. Overall, computational methods and AI could be an integral part of processes vital to human medicine (**Figure 7**). Tendencies to apply these methods are apparent today, even in clinical trials 164.

RNA interference is a potent therapeutic path especially suitable for undruggable targets. However, it faces multiple challenges, like systemic toxicity and obstacles with delivery to tissues other than kidney or liver 165. It has been shown that encapsulation of RNAi molecules with TKIs is able to sensitize resistant cells against TKI therapy 114, 117. Therefore, it possesses a promising future perspective for combination cancer therapy.

## Conclusions

Current focus on nanomedicines in cancer research stems from their unparalleled characteristics, starting with increased bioavailability that is especially important for poorly soluble drugs, prolonged blood circulation, passage through biological barriers, tumor targeting, improvement of drug efficacy, ability to effectively deliver multiple drugs at once and exploitation of their own physical attributes - optical, electrical or magnetic - to convey visualization, localization, additional therapeutic effect or controlled drug release. These features will be the foundation of future cutting-edge applications ranging from specific tissue targeting, real-time imaging and theranostic approach, hopefully leading to personalized cancer therapy.

All in all, even though TKIs are fundamentally imperfect as any other type of cancer therapy, and there is a long way to reach clinical trials with currently researched TKIs-NPs combinations, there is a great potential in this union - potential in improving patients' quality of life and efficacy of TKI therapy. Attention should be paid to developing NPs with sound safety profiles that avoid damage caused by systemic exposure of the nanocarrier itself. NPs that will stand their ground in the clinical settings and that will be easy to handle by patients themselves, stable and prone to slight deviations from timely medication adherence.

## Figures and Tables

**Table 1:** Current classification of TKIs

Class	Binding	ATP-competitive	Reversible binding	Examples of FDA approved TKIs
Type I	In the ATP-binding pocket of the active enzyme conformation	Yes	Yes	Cabozantinib, Dasatinib, Erlotinib, Gefitinib, Vandetanib,
Type I ½ A	In the ATP-binding pocket of a conformation with DFG-Asp in and αC-helix out with extension to back cleft of the ATP site	Yes	Yes	Lenvatinib, Lapatinib
Type I ½ B	In the ATP-binding pocket of a conformation with DFG-Asp in and αC-helix out without extension to back cleft of the ATP site	Yes	Yes	Alectinib, Ceritinib, Crizotinib, Erlotinib
Type II A	In the ATP-binding pocket of a conformation with DFG-Asp out with extension to back cleft of the ATP site	Yes	Yes	Axitinib, Imatinib, Nilotinib, Ponatinib, Sorafenib
Type II B	In the ATP-binding pocket of a conformation with DFG-Asp out without extension to back cleft of the ATP site	Yes	Yes	Bosutinib, Sunitinib, Nintedanib
Type III	Allosterically next to but outside of ATP-binding site with the extension to back cleft of the ATP site	No	No	-
Type IV	Allosterically not next to the ATP-binding site	No	No	-
Type V	Bivalently to two regions of PK domain	Variable	Yes	-
Type VI	Covalently to Cys residues	No	Usually no	Afatinib, Ibrutinib

**Table 2:** Summary of FDA approved small molecule TKIs according to the type of their target as of 19 March 2020; *- non-receptor TKs

Type of primary target	TKIs
ALK	Alectinib, Brigatinib, Ceritinib, Crizotinib, Entrectinib, Lorlatinib
AXL	Sunitinib
BCR-Abl*	Bosutinib, Dasatinib, Imatinib, Nilotinib, Ponatinib, Regorafenib
BTK*	Acalabrutinib, Ibrutinib
c-Met	Cabozantinib, Crizotinib
DDR family	Nilotinib
EGFR family	Afatinib, Brigatinib, Dacomitinib, Dasatinib, Erlotinib, Gefitinib, Lapatinib, Neratinib, Osimertinib, Vandetanib
Ephrin-like	Cabozantinib, Dasatinib, Vandetanib
FRK family	Vandetanib
Insulin/Insulin-like GF	Brigatinib, Ceritinib, Crizotinib, Entrectinib
JAK family*	Baricitinib, Fedratinib, Ruxolitinib, Tofacitinib, Upadactinib
PDGFR α/β	Axitinib, Gefitinib, Imatinib, Lenvatinib, Nintedanib, Pazopanib, Regorafenib, Sorafenib, Sunitinib
RET	Cabozantinib, Lenvatinib, Regorafenib, Sorafenib, Sunitinib, Vandetanib
Src family*	Bosutinib, Dasatinib, Ponatinib, Vandetanib
SYK	Fostamatinib
TRK family	Cabozantinib, Entrectinib, Larotrectinib
VEGFR family	Axitinib, Brigatinib, Cabozantinib, Dasatinib, Erdafitinib, Fedratinib, Gilteritinib, Imatinib, Lenvatinib, Midostaurin, Nilotinib, Nintedanib, Regorafenib, Pazopanib, Pexidartinib, Ponatinib, Regorafenib, Sorafenib, Sunitinib, Vandetanib.

**Table 3:** List of TKI-nanoconstruct applications published recently

Nanomaterial	TKI	Effect	Targeting	Targeting ligand	Ref.
Gold NPs	Afatinib	Improvement of efficacy and biocompatibility	Passive	-	86
Immunoliposomes	Afatinib Cetuximab	Protection from binding to hemoglobin, strongly enhanced drug delivery and anti-tumor efficacy, selectivity and potentially fewer side effects.	Active	Anti-EGFR antibody	80
Liposomes	Afatinib	Improved anti-tumor activity	Passive	-	166
Colloidal polyethylene glycolated (PEG) gold NPs	Afatinib	Higher cellular uptake, 5 and 20 times more potent than Afatinib alone	Passive	-	167
Cyclic arginylglycylaspartic acid (cRGD) and PEG-modified liposomes	Apatinib	Significant tumor treatment targeting ability, better inhibition of tumor growth, and less toxicity.	Active	cRGD	91
Enzyme responsive size-changeable gold NPs	Cediranib	Enhanced tumor vascular permeability, significant therapeutic effect	Passive	-	168
Poly(lactic-co-glycolic acid) (PLGA)-PEG NPs	Cediranib Verteporfin	Combination drug therapy with phototherapy resulted in significant *in vitro* cytotoxicity.	Passive	-	169
Poly(styrene-*co*-maleic acid) micelles	Crizotinib Dasatinib	Enhanced drug activity of drugs in combination, same anti-proliferative effect *in vitro* as free drug, potent anti-proliferative effect *in vivo.*	Passive	-	170
Human serum albumin (HSA) NPs	Dasatinib	As effective as free drug, reduced endothelial hyperpermeability	Passive	-	171
Poly-L-lactic acid(PLA) NPs modified with polyethyleneimine	Dasatinib Trastuzumab	Better *in vitro* efficacy and sustained release of dasatinib	Active	Anti-HER2 antibody	172
Poly(Cyclohexene Phthalate) NPs	Dasatinib	Superior *in vitro* efficacy	Passive	-	173
PLGA NPs	Dasatinib	Compared to free drug enhanced inhibition of proliferative vitreoretinopathy related cellular contraction.	Passive	-	174
PLGA-conjugated gold NPs	Dasatinib	Enhanced growth inhibition *in vitro* and bioavailability *in vivo*	Passive	-	175
Magnetic micelles	Dasatinib	Increased *in vitro* cytotoxicity and decreased cellular migration	Active	Lactoferrin	176
CdSe/ZnS quantum dots	Desmethyl Erlotinib	Cytotoxic enhancement	Passive	-	177
Nanoparticular platform utilizing fat and supercritical fluid	Erlotinib	Improved water solubility	Passive	-	178
Magnetic iron oxide NPs	Erlotinib	Enhancement of therapeutic efficacy, MRI visualization	Passive	-	142
Nanocrystals formulation	Erlotinib	Solubility and drug efficacy enhancement	Passive	-	179
Folate-conjugated thermosensitive O‑maleoyl modified chitosan micellar NPs	Erlotinib	Significantly enhanced cytotoxicity	Active	Folate	180
Solid lipid NPs	Erlotinib	Higher anticancer activity than free drug	Passive	-	181
Nanoparticulation platform utilizing fat and supercritical fluid	Erlotinib	More potent in inhibiting EGF signaling and in suppressing tumor cell proliferation.	Passive	-	182
Cyclodextrin nanosponge	Erlotinib	Increase of solubility, dissolution and oral bioavailability, higher cellular uptake and *in vitro* cytotoxicity.	Passive	-	183
Polyamidoamine dendrimers	Erlotinib Survivin shRNA Chloroquine	Promoted drug delivery and enhanced drug efficacy	Active	Anti-EGFR aptamer	114
Anti-EGFR aptamer-modified liposomal complexes	Erlotinib O_2_	Superior anti-tumor activity, significant inhibition of cell proliferation and improved apoptosis induction.	Active	Anti-EGFR aptamer	105
Eudragit^®^ RL100	Gefitinib	Enhanced oral bioavailability	Passive	-	184
Human heavy chain apoferritin	Gefitinib	Enhanced anti-tumor activity against HER2 overexpressing SKBR3 breast cancer cell line; decreased uptake in cell line, which does not express HER2.	Passive	-	185
Gold colloidal NPs	Gefitinib	Greater cytotoxicity	Passive	-	186
Gelatin tri-block NPs	Gefitinib Cetuximab siRNA	Effective targeting and high bioavailability, very specific for KRAS G12C	Active	Anti-EGFR antibody	117
PEG-PLA NPs	Gefitinib Cyclosporin A	Improvement of drug efficacy, sensitization of gefitinib resistant cells	Passive	-	187
Chitosan NPs	Gefitinib Chloroquine	Potential to overcome acquired resistance and improve cancer treatment efficacy.	Passive	-	106
Anti‐PD‐L1‐modified liposomal system	Gefitinib Simvastatin	Remodeling the tumor microenvironment, reversing gefitinib resistance and enhancing EGFR T790M‐mutated NSCLC treatment outcomes.	Active	Anti-PD-L1 nanobody	121
Sialic acid-stearic acid conjugate modified on the surface of nanocomplexes	Ibrutinib	Suppressed tumor progression	Active	Sialic acid	188
HSA NPs	Imatinib base	35% greater cytotoxicity	Passive	-	189
Galactoxyloglucan NPs	Imatinib mesylate	Enhancement of cytotoxic potential and reversal of multidrug resistance	Passive	-	190
PLGA NPs	Imatinib mesylate	Improved cytotoxic compared to free drug, 28 day‑long oral administration showed no significant cardiotoxicity or associated changes.	Passive	-	87
Poly(ε-caprolactone) NPs with chitosan	Imatinib mesylate	Improved drug's kinetics and efficacy, long-lasting inactivation of BCR-ABL autokinase activity.	Passive	-	191
Polycaprolactone nanocapsules	Lapatinib	Improvement of anti-tumor effects	Passive	-	192
Hyaluronic acid-D-α-tocopherol succinate-(4-carboxybutyl)triphenyl phosphonium bromide-based NPs (HA-TS-TPP)	Lapatinib	Better tumor growth suppression, triple negative breast cancer targeting	Active	HA; TS; TPP	193
HSA NPs	Lapatinib	Enhanced cell cytotoxicity and induction of apoptosis, inhibition of HER2 phosphorylation and superior anti-tumor efficacy *in vivo*, no subchronic toxicity within 60 days of treatment.	Passive	-	88
HSA NPs	Lapatinib	Inhibition of adhesion, migration and invasion ability of cells more effectively; extension of median survival time in mice.	Passive	-	89
HSA NPs	Lapatinib	Increased accumulation of Lapatinib in tumor tissue, better suppression effects both on primary breast cancer and lung metastasis *in vivo.*	Passive	-	194
PTX NPs and LAPA microparticles in a thermosensitive hydrogel	Lapatinib Paclitaxel	Synergistic effect of LAPA and PTX on cell line overexpressing HER2 and P-gp; significantly less nonspecific toxicity.	Passive	-	115
Liposomes	Ponatinib	Significant tumor growth inhibition (by 60.4%) and markedly reduced side effects.	Passive	-	195
Liposomes	Nintedanib	Significant tumor growth inhibition (by 60.4%) and markedly reduced side effects.	Passive	-	195
Linear-dendritic self-assembling polymeric drug carrier release-triggered by enzyme Cathepsin B	Saracatinib	Better suppression of metastasis	Passive	-	196
Reduced graphene oxide nanosheets	Sorafenib	Improved cytotoxicity	Passive	-	197
Lipid nanocapsules	Sorafenib	Early tumor vascular normalization, decreased proliferation	Passive	-	198
Focused ultrasound-triggered thermosensitive liposomes	Sorafenib	Significantly lower cell viability	Passive	-	90
Self-assembling PEG-vitamin E succinate derivative NPs	Sorafenib Curcumin	Enhanced *in vitro* cytotoxicity and anti-angiogenesis, greater drug concentration in organs *in vivo* and inhibition of tumor growth.	Passive	-	132
HSA encapsulated gold nanorods paired with photothermal ablation	Sorafenib	100% tumor cell kill rate	Passive	-	199
Irradiated HSA gold nanorods	Sorafenib	Significantly induced hyperthermia, enhanced cytotoxicity	Passive	-	200
PLA-PEG-poly(L)-lysine-diethylenetriamine pentaacetic acid NPs with gadolinium and poly(L-histidine)-PEG-biotin modification	Sorafenib	Improved diagnostic abilities, higher anti-tumor effect *in vitro* and *in vivo*.	Active	Anti-VEGFR antibody	141
Styrene-*co*-maleic acid micelles	Sorafenib Nilotinib	Greater cytotoxicity, decreased cell proliferation, increased apoptosis relative to the free TKIs.	Passive	-	110
Lactobionic acid modified and pH-sensitive chitosan-conjugated mesoporous silica nanocomplex	Sorafenib Ursolic acid	Enhanced bioavailability, synergistic cytotoxicity, significant increase of cellular apoptosis and down-regulation of EGFR and VEGFR2 proteins expression, significant reduction of tumor burden in hepatocellular carcinoma.	Active	Lactobionic acid	122
Integrin-targeted cAmpRGD liposomes	Sunitinib	Inhibition of growth and adhesion, anti-angiogenic effect	Active	cAmpRGD	201
Self-nanoemulsifying drug delivery system	Sunitinib	Bioavailability and cytotoxicity increase	Passive	-	79
PLGA-PEG-MBA polymeric micelles combined with mannose-modified lipid calcium phosphate NPs-based Trp2 vaccine	Sunitinib	Abrogation of tumor-associated immune suppression, enhanced therapeutic efficacy.	Passive	-	119
Self-nanoemulsifying drug delivery system	Sunitinib malate	Two-fold increase in efficacy	Passive	-	202
PEG-NLG919-based immunostimulatory nanocarrier	Sunitinib Paclitaxel NLG919	More active tumor immune microenvironment and further improved anti-tumor activity.	Passive	-	203
BSA-coated superparamagnetic iron oxide NPs	Sunitinib Curcumin	Significant tumor inhibition yet least drug-induced toxicity both *in vitro* and *in vivo* when compared with free drug formulations.	Passive	-	104
iRGD-PEG-PLA NPs	Vandetanib	More effective cytotoxic activity *in vitro* and tumor inhibition *in vivo*	Active	iRGD	204
Micellar gold NPs	Vandetanib	Inhibition of tumor growth	Passive	-	205

## Abbreviations

AUC: area under curve
ATP: adenosine triphosphate
BBB: blood-brain barrier
CTCAE: common terminology criteria for adverse events
EPR: enhanced permeability and retention
FA: folic acid
i.v.: intravenous
MRI: magnetic resonance imaging
NP: nanoparticle
PK: protein kinase
PPI: proton pump inhibitor
PTB: phosphotyrosine-binding
RTK: receptor tyrosine kinase
SH2: Src homology-2
SNEDDS: self-nanoemulsifying delivery system
TK: tyrosine kinase
TKI: tyrosine kinase inhibitor
